# Periodic forces trigger knot untying during translocation of knotted proteins

**DOI:** 10.1038/srep21702

**Published:** 2016-03-21

**Authors:** Piotr Szymczak

**Affiliations:** 1 Institute of Theoretical Physics, Faculty of Physics, University of Warsaw, Warsaw, Poland

## Abstract

Proteins need to be unfolded when translocated through the pores in mitochondrial and other cellular membranes. Knotted proteins, however, might get stuck during this process, jamming the pore, since the diameter of the pore is smaller than the size of maximally tightened knot. The jamming probability dramatically increases as the magnitude of the driving force exceeds a critical value, *F*_*c*_. In this numerical study, we show that for deep knots *F*_*c*_ lies below the force range over which molecular import motors operate, which suggest that in these cases the knots will tighten and block the pores. Next, we show how such topological traps might be prevented by using a pulling protocol of a repetitive, on-off character. Such a repetitive pulling is biologically relevant, since the mitochondrial import motor, like other molecular motors transforms chemical energy into directed motions via nucleotide-hydrolysis-mediated conformational changes, which are cyclic in character.

While struggling with tangled earphones, garden hoses, or extension cords we feel that the world would be a better place without knots. However, not all knots are useless, some are handy or even life-saving, as any sailing or rock-climbing aficionado will tell you. Since any chain long enough have a tendency to get knotted^1^,^2^,^3^ one finds knots also at the microscale - in DNA^4^, and even in proteins^5^,^6^,^7^. Although some of these knots might have functional importance, providing the additional stability necessary for maintaining the global fold and function of proteins under harsh conditions^8^,^9^, they can also be problematic for the cell. Knots in DNA could lead to blocking of its replication and transcription^10^,^11^, and therefore need to be quickly removed, which is accomplished by topoisomerases. In proteins, knots were hypothesized to affect the ability of the molecules to be degraded in proteasome^6^,^12^ or translocated through the intercellular membranes, e.g. during import into mitochondria^13^,^14^. It is estimated that more than 50% of the proteins produced in cells must traverse cellular membranes, thus translocation is vital for functioning of the cell^15^,^16^. In these processes, the proteins have to pass through constrictions that are too narrow to accommodate folded structures, thus translocation must be coupled to protein unfolding^15^,^17^,^18^,^19^,^20^. However, as shown in a number of theoretical and experimental studies, the protein knots get tightened under the tension. The radius of gyration of the tight knot, 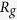, is about 7–8 Å, whereas the diameters of the narrowest constriction of the mitochondrial pores or proteasomal openings are in the 12–15 Å range^21^, i.e. smaller than 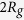, thus knots are sterically prevented from entering the pore. The translocation would therefore be halted, unless the protein succeeds in sliding the knot off during the translocation.

In this paper, through Brownian dynamics simulations of knotted protein translocation we show that knot tightening probability strongly depends on the force with which the protein is pulled into the pore. In particular, it is demonstrated that there exists a critical force, 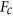, above which the tightening becomes almost certain. For deep knots (with more than 30 aminoacids between the end of the knotted core and the free end of the protein) 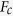 is shown to lie below the force range over which molecular import motors operate, which suggest that in these cases knots will tighten and block the pores. Next, we show how such topological traps might be prevented by using a pulling protocol of a repetitive, on-off character. Such a repetitive pulling is biologically relevant, since molecular import motors are ATP-hydrolysis driven and thus cyclic in character.

**The model of the protein and the pore.** Despite the rapid increase in computer power, the computational demands are still a barrier, preventing atomistically detailed simulations of the translocation process, due to the large system sizes and long timescales involved. This motivated the use of coarse-grained model of both the protein and the pore in the present study. For protein, we adopt a G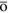-type model, in which individual amino acids are replaced by beads of uniform size placed at the locations of the *C*_*α*_ atoms. The effective potential of the interaction between these beads is tailored to give the lowest energy to the native state of a protein. A particular implementation of the Gō-type model followed here is by Cieplak and co-workers^9^,^22^. In short, the protein structure is represented by a chain of *C*_*α*_ atoms tethered along the backbone by harmonic potentials with minima at 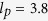 Å. Effective interactions between residues are split into native and nonnative interactions by checking for overlaps between the enlarged van der Waals surfaces of the residues^23^. Amino acids (i and j) that overlap are endowed with the effective Lennard-Jones potential 
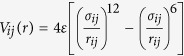
 with energy scale *ε* and pair-by-pair distances *r*_*ij*_. The length parameters, 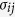, are chosen such that the potential minima correspond pair by pair to the native state distance between the residues. Nonnative contacts are represented by hardcore repulsion to prevent entanglements. Correct chirality is imposed by the angle-dependent term in the Hamiltonian. A standard Brownian dynamics algorithm is used to move the residues, at the temperature corresponding to 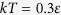, which is the optimal folding temperature for this coarse-grained model^24^. The characteristic timescale 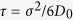 is set by the time it takes for the amino acid to diffuse the typical contact distance 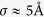. Next, the pore is modeled as a cylindrical structure (with axis aligned with the positive z-axis) interacting with the amino acids by the potential 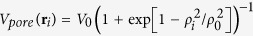 (for *z*_*i*_ > 0), as proposed by J.M. Deutsch^25^. Here 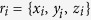 is the position of ith aminoacid, *z* is oriented along the pore axis and 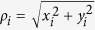 is the distance from the axis. The potential is small within the radius 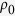 from the axis of the pore and then rises sharply. Additionally, to prevent the protein from entering the membrane except through the pore, a short range, repulsive membrane potential is introduced at its outer side with 
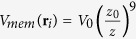
 (for *z*_*i*_  < 0, 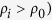. In the simulations reported here, 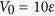, 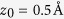 and 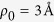. Note that the pore potential acts on the centers of the particles. Since the van der Waals radii of amino acids are in the range of ~3.5–4.5 Å, the above value of 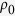 corresponds to the effective pore radius of about 6.5–7.5 Å, which is consistent with the values reported for the narrowest constriction in the mitochondrial pores^21^. The question of mapping the force-scales and time scales between the simulation and experiment is rather subtle, with the result depending on which experimental quantity is used to set the scale. Comparison of diffusion coefficients of the model proteins with the experimental values leads to an estimate 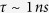
26. On the other hand the comparison of the protein folding times in the model and *in vivo* leads to 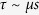 (e.g. ubiqutin in silico folds on the timescale of 10^3^ *τ*^27^ whereas *in vivo* - it is in tens of millisecond range^28^). Such a discrepancy is connected with a relatively small frustration of the Gō-type protein models^29^. As for the forces, the conversion between experimental and numerical values is provided by the correlation of the maximum resistance force in constant velocity pulling. The comparison of the forces measured in AFM experiments with those recorded in the numerical simulations for 28 different proteins have shown a good correlation (with Pearson correlation coefficient of 0.89) provided that the force unit in the model, *ε*/Å, is translated into about 70*pN*^24^,^30^.

## Results and Discussion

### Translocation simulations

**Figure 1:**
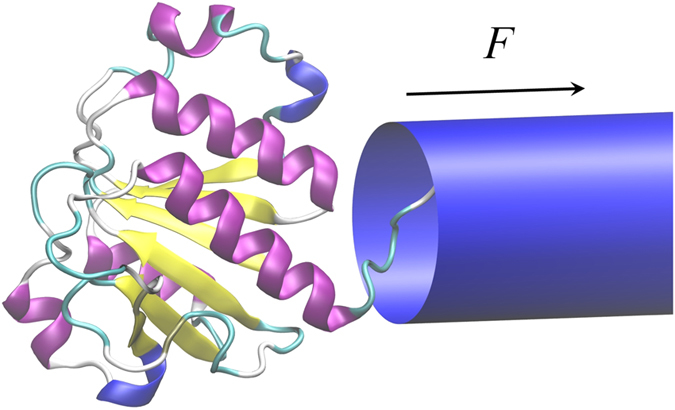
The simulation setup: the protein (here 1j85) and the pore (blue).

**Figure 2:**
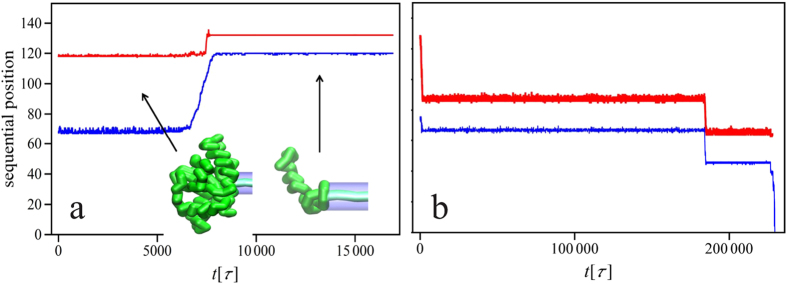
The movement of knotted core during the translocation of the protein 1ns5 pulled into the pore by the N terminus with *F* = 2.2*ε*/Å (a) and with the force *F* = 1.4*ε*/Å by the C terminus (b). The colors mark the two ends of the knot as they move along the chain. In (**a**) the knot gets tightened and blocks the pore, whereas in (**b**) - it slides off the chain. The insets of (**a**) show the conformations of the protein backbone at the beginning of the translocation and at jamming. The final position of the knot is between the aminoacids 119 and 134. Only a portion of a longer trajectory (up to 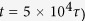 trajectory is shown in panel (**a**), however no further changes in the knot position were observed beyond 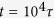.

A schematic illustration of the simulation setup is presented in Fig. 1. At the beginning of the simulations the protein in its native conformation is placed at the outer side of the membrane near the pore entrance. In the cell, the transport of proteins into mitochondria is usually mediated by a loosely folded presequence, which is modeled here as a loose piece of a peptide chain (10 amino acid long). One end of the presequence is attached to the protein terminus (N or C), while the other end is pulled with the force 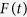 along the pore axis. The force is either constant in time or switched on and off with the period *T*. Several knotted proteins were studied, as summarized in Table 1. During the simulation we not only record the conformation of the protein but also track the position of the knotted core, i.e. the smallest region that will remain knotted when the residues are successively deleted from both ends^8^. Thereby we obtain the trajectories of knot’s ends in the sequential space, such as those shown in Fig. 2.

The simulation setup is similar in spirit to some of the previous models of protein translocation^13^,^14^,^31^,^32^,^33^,^34^,^35^,^36^,^37^,^38^. In particular, West *et al*. have studied differences and similarities between the AFM-induced protein stretching and the translocation-induced unfolding, finding that in the latter process the protein structures near the rim of the pore are responsible for the overall mechanical stability of a molecule. In an important series of papers^31^,^33^,^39^,^40^, Makarov and co-workers have explored the energy landscape associated with translocation and found it to be significantly different from that characterizing a simple AFM stretching.

These results were complemented by the studies of Cecconi *et al*.^36^,^37^,^41^ who have related the free-energy landscape of protein translocation to the mean translocation time, identifying the bottlenecks of the transport. Importantly, Huang and Makarov have also studied the translocation of the knotted polypeptide chains^34^, which was shown to proceed through multiple slippage events suggesting a rugged energy landscape with multiple metastable minima. The presence of a knot was found to considerably increase the translocation time, an effect increasing with knot complexity. However, in contrast to the present study and the simulations reported in^13^,^14^ the parameters of the pore allowed the knotted chain to enter inside it, thus jamming was not possible. Nevertheless, as mentioned above, careful experimental studies on the size of the tight protein knots^12^ supported by both all-atom^42^ as well as coarse-grained MD simulations^9^ put the size of the smallest proteinic knots at 7.2 Å, i.e. too large to enter either the mitochondrial pore or proteasome opening. Thus, as it was demonstrated in^13^,^14^ two outcomes are eventually possible: either the knot moves towards the end of the chain and simply slides away, or it gets tightened and jams the opening. Representative examples of the trajectories of the knot’s ends in these two cases are presented in Fig. 2, with the left panel (as well as movie S1 in the SM) illustrating the jamming of protein 1ns5 pulled into the pore by the N terminus. Contrastingly, the right panel presents the situation in which the same protein is pulled by the C terminus and the knot slides off the chain. The most striking feature of the motion of the knot under the force is that it is a collection of successive jumps over multiple energy barriers, interspersed with waiting periods in metastable states. As elucidated in^9^ this is because one or both of the knot’s ends get pinned on sharp turns of a protein backbone. During successive jumps the knot invariably shrinks in size until it gets fully tightened, reaching the size of 12–15 aminoacids (for a trefoil knot), or slides off the chain. Once the knot gets fully tightened, we never observe it to loosen up again, at least in the force range studied.

### Macroscopic analogy

**Figure 3:**
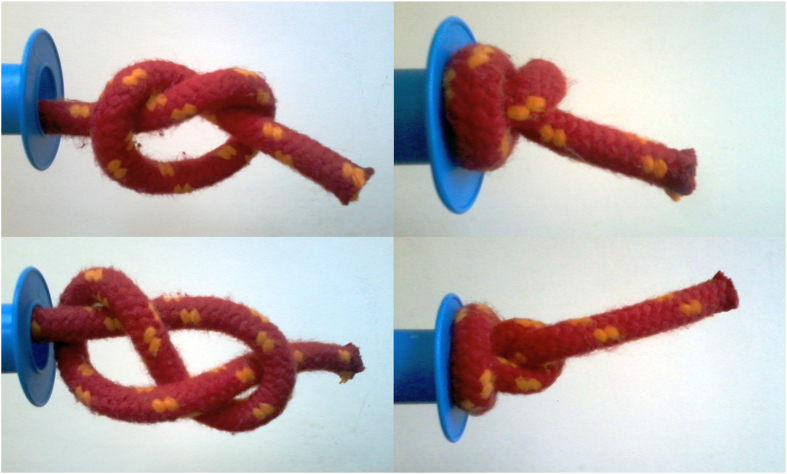
Pulling the knotted rope through a model pore. Trefoil (upper left) and figure-of-eight knot (lower left) on the climbing rope is tugged sharply into the spool opening, which results in tightening of the knot (right panels).

A macroscopic analogy can be helpful here - let us tie a loose knot on a piece of rope and pull it through a cylindrical hole (*cf*. Fig. 3). If pulled sharply, the knot invariably tightens; however if tugged slowly - it might be able to slide towards the free end of the rope and then get untied. There are several things to be learned from such a macroscale rope experiment. First of all, an important factor in knot self-tightening is friction^43^: if the friction coefficient is large enough, then the knot, once tightened, will hold no matter what force is applied: tugging at the rope will only increase the normal forces squeezing the two pieces of the rope together which results in larger frictional forces preventing the slippage. Moreover, the forms of the knot tightened around the pore opening are similar at macro- and micro-scale (*cf*. Figs 2 and 3) - they both involve a fastened loop around the entrance of the pore. And yet the rope analogy holds only to a limited degree, mostly because it neglects the role of thermal fluctuations: whereas the rope, once tightened, stays tight, a protein knot might be spontaneously loosened due to the Brownian motion. Additionally, proteins - in contrast to ropes - are highly nonhomogeneous, with an intricate network of contacts between different amino acids. This inhomogeneity was shown to be important for the knot dynamics, even in the absence of the pore: as the knotted core shrinks in the process of tightening, one or both of the knot’s ends get pinned on sharp turns of a protein backbone^9^. This effect is even more dramatic during the translocation, with sharp turns acting as potential pinning centers which can block the backbone on its way through the pore opening leading to the tightening of the knot and blocking of the pore^14^. This is why deep knots (with more than 30 aminoacids between the end of the knotted core and the free end of the protein) can easily jam the pores. We note that in this context whether the knot can be called “shallow” or “deep” depends on the pulling direction - if the protein is pulled e.g. by the N terminus, then the distance between the end of the knot and the *C* terminus determines its behaviour.

### Jamming probability is force-dependent

**Figure 4:**
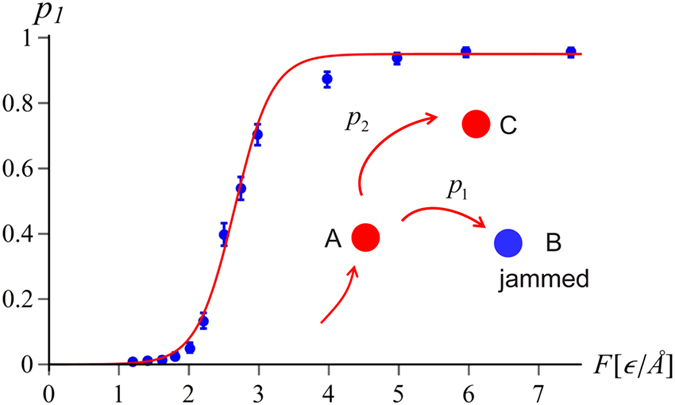
Jamming probability as a function of force for the protein 2k0a pulled by the N terminus. The data points in this and other figures have been obtained based on the average over 100 simulations runs. The red line represents the fit to the two-pathway model (1) with 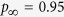, 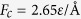 and 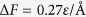. The inset shows schematically the kinetic partitioning between the translocation 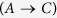 and knot tightening 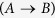. Error bars mark 68% Wilson confidence intervals^73^.

As illustrated in Fig. 4, the probability of knot tightening during the translocation (marked 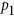 in the inset) rapidly increases with the force. The sigmoidal shape of 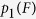 dependence is consistent with the presence of two alternative pathways, one leading to the tightening 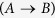, the other - to the translocation 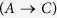. Such pathways have been observed previously in the knot tightening dynamics^9^, and - at least for moderate forces - they were shown to follow the Bell’s model^44^, with the relative probability of tightening given by 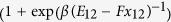, where 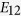 and 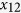 is the difference in energy barrier heights and locations of transition states, respectively. However, we observe that in the large force limit the probability of jamming of the pore asymptotes to a constant different from 1. We hypothesize that this behaviour is connected with the form of the underlying energy landscape. Namely, at high forces the kinetic barriers disappear and both states (B and C) find themselves lower than A on the energy landscape, forming so-called valley-ridge inflection point^45^,^46^. In such a situation - where a pathway forks into two downhill routes with a dynamically unstable ridge separating them, the relative probability of ending up in a particular state depends on the tiny details of the initial conformation, which are largely determined by thermal fluctuations and not by the force. A simplest model taking this asymptotics into account would give the jamming probability of the form


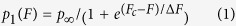


where 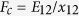 is a critical force at which the jamming transition occurs whereas 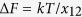 measures the width of the transition region. As illustrated in Fig. 4 the simulation data are well-fitted by Eq. (1), which confirms the applicability of the Bell’s model in the description of the tightening dynamics. However, within the studied proteins, in three cases only 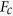 was found to lie within the numerically accessible force regime (*F* > 1*ε*/Å). This takes place for relatively shallow knots. For deep knots, critical forces are lower and the simulations are probing large-*F* tail of the distribution only. Unfortunately, collecting statistically meaningful data in low force regime (*F*  < 1*ε*/Å) is difficult due to long translocation times involved. On the other hand, molecular motors importing the proteins act with the forces of the order of 30pN^47^, which corresponds to 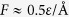 in the present context. By fitting (1) to the simulation data we can nevertheless estimate the critical forces 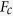 also in these cases, as quoted in Table 1. Relatively large errors of these values are related to the difficulty in estimating the parameters of Eq. (1) from the tail of the distribution. Nevertheless, this data suggests that there is a group of proteins for which the critical jamming forces are in 3–10 *pN* range, significantly below the forces relevant for the import motors. Importantly, the decrease of the pulling force beyond 20–30pN is not a viable option for the import machinery, since lower forces will not be able to denature protein structures over biological time scales. Shallow knots have much larger 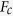 and they will translocate easily when pulled by molecular motors. There is also a group of very shallow knots which never get stuck - in these cases the stretch of the protein backbone between the knot’s end and the terminus does not contain any sharp turns.

Jamming of the translocating knotted polymers at high pulling forces has first been reported by Rosa *et al*. in the simulations of polyelectrolyte chains^48^. The differences between the knot tightening in homo- and heteropolymers are elucidated in^49^, where it is in particular shown that only in the latter case a complete self-tightening of the knot can be achieved. This is confirmed by the results of this study, where we find that once the knot tightens it becomes completely immobilized, until the force is relaxed. Conversely, Rosa *et al*. report that even at the highest forces considered there, the effective mobility of the chain remains nonzero, and the chain still translocates, albeit at a much decreased rate.

Interestingly, no jamming is observed in the passive ejection of DNA out of a spherical cavity, e.g. virial capsid^50^,^51^. In the most recent study of these systems Marenduzzo *et al*.^52^ emphasize the importance of cholesteric interactions in a DNA ejection problems. The ordering effect of such interactions leads to the prevalence of torus knots in the capsid, which unravel gradually by simplifying their topology in a stepwise fashion. Although occasionally the stochastic movement of the chain inside the capsid leads to a spontaneous formation of more complicated knots which can stop the ejection, in most cases the knotted chain is able to exit the capsid without getting jammed.

### Periodic forces trigger knot untying during translocation of knotted proteins

**Figure 5:**
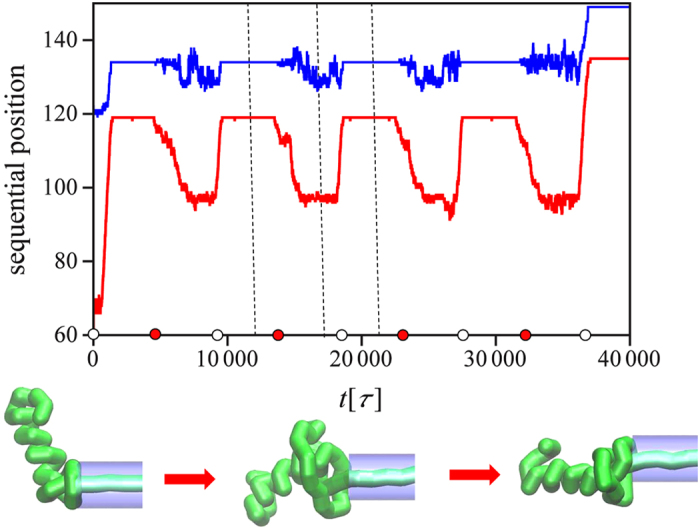
The movement of knotted core during the translocation of the protein 1ns5 pulled into the pore by the N terminus with an on-off cycle of 4500*τ* when the force is switched between *F*  = 4*ε*/Å in on-state and *F* = 0 in off-state. The colors (red and blue) mark the two ends of the knot as they move along the chain. The circles on the time axis represent the moments of switching the force on (white) and off (red). The conformations below correspond to the time moments marked by the dashed lines.

How does nature gets around the topological traps in the protein translocation? Our numerical results seem to suggest that a very efficient way of avoiding pore jamming is by using the forces of cyclic, on-off character. In fact, molecular motors never operate in a continuous way, since they are fed by the ATP hydrolysis. To perform a net work during a hydrolysis cycle, a motor undergoes a conformational change (a power stroke) while bound to the substrate, followed by a recovery stroke while detached^53^,^54^. Since the force exerted during the power-stroke is nearly constant^55^, the simplest model of the motor action involves a force acting in an off-on manner: for the first half of the period it is 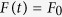, whereas for the second 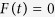. The effect of such a cyclic force on the translocation is shown in Fig. 5 and Movie S2. At first, when the force is on, the knot tightens in analogy with Fig. 2a. However, as the force is switched off, the protein relaxes and the knot swells in a process similar to “knotted core breathing” mechanism described first in^56^ - local length fluctuations in exchange with the knot’s immediate vicinity slightly change its size, allowing diffusive motion of the knot along the chain. After 10^3^–10^4^ *τ* the knot relaxes from the tightened state and finds itself in a vicinity of a previous intermediate state (*A*). In the next force cycle, the protein again attempts to translocate through the pore. A mechanism by which the repetitive pulling allows to avoid the topological traps is thus similar in spirit to that reported by Tian and Andricioaei in the context of avoiding long-lived intermediates during co-translocational unfolding^57^: since the probability of getting trapped in each of *n* successive tries is 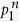, which rapidly decreases with *n*, repetitive trying always leads to a final success.

### Dependence of the translocation time on the force period

**Figure 6:**
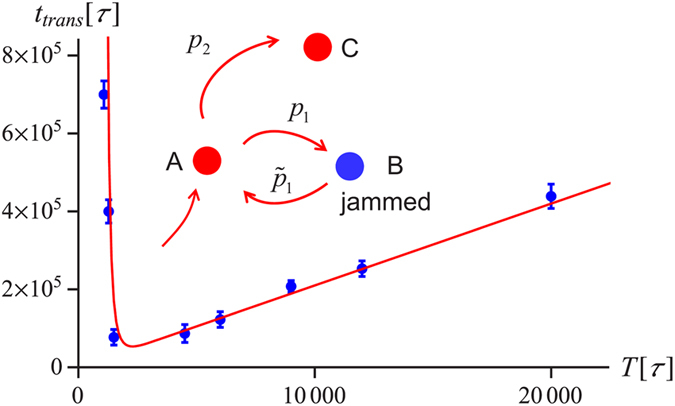
Mean translocation time as a function of the force period for a knotted protein (1ns5) pulled by the *N* terminus. The amplitude of the force is *F*_0_ = 3*ε*/Å. The curve represents Eq. (2), with 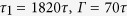 and 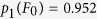, estimated as described in the text. The error bars mark the standard deviation from the mean.

**Figure 7:**
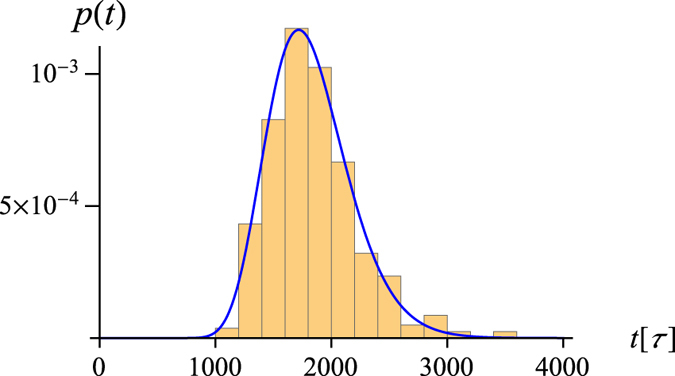
The distribution of knot loosening times for the protein 1ns5 after the force is relaxed. Initially the knot is fully tightened with the knotted core spanning 15 aminoacids (corresponding to the plateaus in Fig. 5) and the final state corresponds to the knotted core spanning ~30 aminoacids (corresponding to the minima of the red curve in Fig. 5). The blue curve is a fit to the inverse Gaussian distribution 
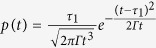
 with 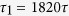 and 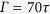.

The dependence of the translocation time 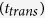 on the period of the force (Fig. 6) shows a clear minimum corresponding to an optimal switching period. This can be rationalized by noting that a successful translocation after a jamming event, needs i) loosening of the knot during the off-force period, i.e. the insertion of some stored length into the knotted core, which allows it to escape from a tightened configuration (*B*) to the previous metastable state (*A*), and ii) a successful translocation to state *C* avoiding the trap in the tightened state (*cf*. the inset of. Fig. 6). The translocation time will then be of the form


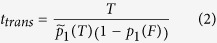


where 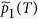 and 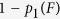 are the probabilities of (i) and (ii) respectively. The former depends on *T* since too short a period makes it impossible for the knot to escape from a basin of attraction of the tightened state. The latter is a function of the force, as given by Eq. (1). Performing a series of simulations starting from the tightened state, we have obtained the distribution of first passage times of the knot on 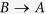 trajectory and found it follow an inverse Gaussian distribution^58^, as shown in Fig. 7. This suggests that the swelling of the knot during the off-force state can be treated as one-dimensional biased diffusion along the coordinate *N*, corresponding to the size of the knotted core.

The bias here comes from the contact interactions between the amino acids, which are trying to refold the protein and is absent in the case of homogeneous chains, where the movement of the knot was shown to be purely diffusive^59^. Treating state *A* as the absorbing boundary, we get the probability that *A* is reached within time *T* as^60^,


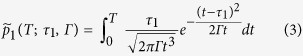


where 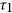 is the mean first passage time and 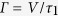 with *V* being the variance. The theoretical estimate of 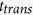 according to (2) with the above-given 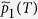 is shown as a solid curve in Fig. 6. Note that the curve is not a direct fit to the data, since 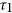 and *V* has been obtained by an independent analysis of the first passage time distribution, whereas 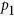 has been obtained by performing a series of translocation attempts starting from *A*.

### Dependence of the translocation time on the force magnitude

**Figure 8:**
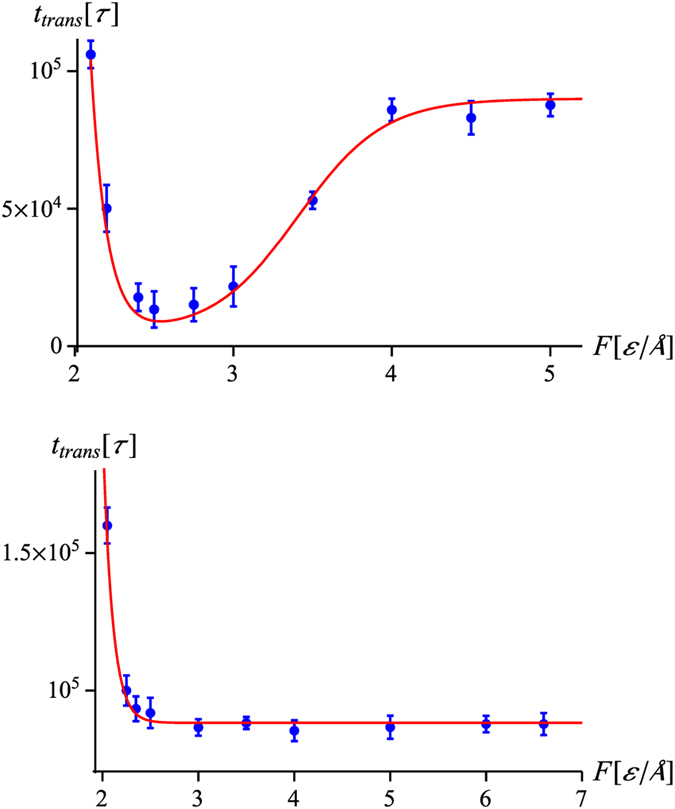
Mean translocation time as a function of the magnitude of the pulling force for repetitive protocol for the protein 2k0a pulled by the *N* terminus (top) and 1ns5 pulled by the *C* terminus (bottom). The period of the force in both cases is equal to 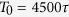. The red curve in the top panel represents the formula 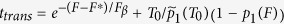, as given by Eq. (4) . The values of the parameters are 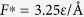, 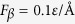, 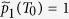, whereas 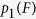 is given by Eq. (1) with 
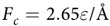
 and 
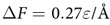
. In the bottom panel, the red curve is given by 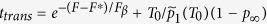, since the forces involved are much larger than *F*_*c*_ for this protein (*cf*. Table 1). The remaining parameters are 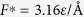, 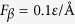, 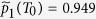 and 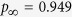. The error bars mark the standard deviation from the mean.

Finally, let us analyze the dependence of the translocation time on the magnitude of the pulling force. As illustrated in Fig. 8, there is an exponential increase of 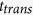 at small forces which is not related to the presence of the knot, but simply to the presence of kinetic barriers in protein unfolding. On the other hand, the increase of 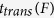 at larger forces and the subsequent plateau visible in the upper panel of Fig. 8 are closely connected with the force-dependent jamming probability (1). Altogether, the 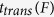 dependence at fixed force period, *T*_0_, is well described by the formula


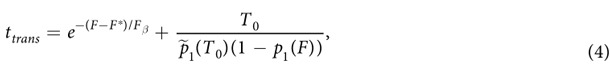


shown by a red line in Fig. 8. Again, this is only a partial fit, since 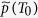 and 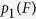 are assessed independently. In the above, 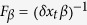 is a characteristic thermal force associated with the main kinetic barrier^61^, with 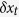 being the position of the respective transition state along the reaction coordinate. Next, *F* * is a threshold force at which the barrier disappears^60^,^62^,^63^. If *F* * < *F*_*c*_, then the 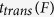 has a form analogous to that in the upper panel of Fig. 8. If, on the other hand, *F* * > *F*_*c*_, then the effects of the first, exponential term in 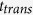 dominate those of the second term and the translocation times monotonically decrease with the force, as illustrated in the lower panel of Fig. 8.

## Summary

To summarize, Brownian dynamics simulations of protein translocation suggest that the pore jamming by tight protein knots can be avoided by the use of a pulling protocol of a repetitive, on-off character. This mechanism is remarkably simple and yet very robust - all of the proteins considered here, no matter how large and complex, will eventually make it through the pore, becoming untied in the process. Importantly, such a repetitive pulling is biologically relevant, since the mitochondrial import motors are cyclic in character.

Experimental verification of these conclusions should be possible. One option would be to fuse a mitochondrial targeting sequence to one of the knotted proteins^64^ and subject it to *in vitro* import into isolated mitochondria, analogously to the experiments on other proteins^65^,^66^. Another possibility is to use modern artificial nanopore technology. Engineered protein nanopores have been successfully used for the detection of DNA chains^67^,^68^, including the knotted ones^69^. Recently, it was demonstrated that they can also be used for the detection of proteins^70^,^71^,^72^. These techniques should provide a means for experimental analysis of the impact of periodic forces on knotted protein translocation.

## Additional Information

**How to cite this article**: Szymczak, P. Periodic forces trigger knot untying during translocation of knotted proteins. *Sci. Rep*. **6**, 21702; doi: 10.1038/srep21702 (2016).

## Supplementary Material

Supplementary Information

Supplementary Information

Supplementary Information

## Figures and Tables

**Table 1:** Proteins considered and the characteristics of their knots as well as critical forces, *F*_*c*_, at which they jam the pores (*cf*. Eq. 1); *N* and *C* stand for pulling by the N and C terminus respectively; *free* means that the protein always translocates.

Protein	pdb	length	knotted core	knot type	*F*_*c*_(*N*)[*ε*/Å]	*F*_*c*_(*C*)[*ε*/Å]
YbeA from E. coli	1ns5	1–153	67–121	trefoil	0.57 ± 0.04	0.15 ± 0.05
zinc-finger motif	2k0a	−1–107	21–73	trefoil	2.65 ± 0.03	free
YibK methyltransferase	1j85	1–156	75–120	trefoil	free	0.2 ± 0.05
YbeA-like (T.maritima)	1o6d	1–147	65–118	trefoil	1.64 ± 0.03	0.16 ± 0.04
Ribbon-helix-helix protein	2efv	6–87	13–80	trefoil	free	free
transcarbamylase (X.campestris)	1yh1	3–336	172–254	trefoil	0.05 ± 0.05	0.1 ± 0.05
FLIN2 chimaeric protein	1j2o(14)	1–114	42–95	figure-of-eight	free	1.95 ± 0.02

## References

[b1] Frank-Kamenetskii M. D., Lukashin A. V. & Vologodskii A. V. Statistical mechanics and topology of polymer chains. Nature 258, 398–402 (1975). 1196369 10.1038/258398a0

[b2] Raymer D. M. & Smith D. E. Spontaneous knotting of an agitated string. Proc. Natl. Acad. Sci. USA 104, 16432–16437 (2007). 17911269 10.1073/pnas.0611320104 PMC2034230

[b3] Sumners D. & Whittington S. G. Knots in self-avoiding walks. J. Phys. A: Math. Gen. 21, 1689 (1988).

[b4] Bates A. D., Maxwell A. DNA Topology (Oxford University Press, 2005).

[b5] Taylor W. R. & Lin K. Protein knots: a tangled problem. Nature 421, 25–25 (2003). 12511935 10.1038/421025a

[b6] Virnau P., Mirny L. & Kardar M. Intricate knots in proteins: Function and evolution. PLoS Comput. Biol. 2, e122 (2006). 16978047 10.1371/journal.pcbi.0020122 PMC1570178

[b7] Millett K. C., Rawdon E. J., Stasiak A. & Sułkowska J. I. Identifying knots in proteins. Biochem. Soc. Trans 41, 533–537 (2013). 23514149 10.1042/BST20120339

[b8] Taylor W. R. A deeply knotted protein structure and how it might fold. Nature 406, 916–919 (2000). 10972297 10.1038/35022623

[b9] Sulkowska J., Sulkowski P., Szymczak P. & Cieplak M. Tightening of knots in proteins. Phys. Rev. Lett. 100, 058106 (2008). 18352439 10.1103/PhysRevLett.100.058106

[b10] Portugal J. & Rodrguez-Campos A. T7 RNA polymerase cannot transcribe through a highly knotted DNA template. Nucleic Acids Res. 24, 4890–4894 (1996). 9016657 10.1093/nar/24.24.4890 PMC146338

[b11] Deibler R. W., Mann J. K., Sumners D. W. & Zechiedrich L. Hin-mediated DNA knotting and recombining promote replicon dysfunction and mutation. BMC Mol. Biol. 8, 44 (2007). 17531098 10.1186/1471-2199-8-44 PMC1904230

[b12] Bornschlögl T. . Tightening the knot in phytochrome by single-molecule atomic force microscopy. Biophys. J. 96, 1508–14 (2009). 19217867 10.1016/j.bpj.2008.11.012 PMC2717258

[b13] Szymczak P. Tight knots in proteins: can they block the mitochondrial pores? Biochem. Soc. Trans. 41, 620–624 (2013). 23514165 10.1042/BST20120261

[b14] Szymczak P. Translocation of knotted proteins through a pore. Eur. Phys. J. Special Topics 223, 1805–1821 (2014).

[b15] Panja D., Barkema G. T. & Kolomeisky A. B. Through the eye of the needle: recent advances in understanding biopolymer translocation. J. Phys.: Condens. Matt. 25, 413101 (2013). 10.1088/0953-8984/25/41/413101 24025200

[b16] Muthukumar M. Polymer Translocation (CRC Press, 2011).

[b17] Pfanner N. & Meijer M. Pulling in the proteins. Curr. Biol 5, 132–135 (1995). 7743175 10.1016/s0960-9822(95)00033-9

[b18] Matouschek A., Pfanner N. & Voos W. Protein unfolding by mitochondria. EMBO Rep. 1, 404–410 (2000). 11258479 10.1093/embo-reports/kvd093 PMC1083766

[b19] Mokranjac D. & Neupert W. Protein import into mitochondria. Biochem. Soc. Trans. 33 1019–1023 (2005). 16246036 10.1042/BST20051019

[b20] Muthukumar M. Macromolecular mechanisms of protein translocation. Protein Pept. Lett. 21, 209–216 (2014). 24370256 10.2174/09298665113209990079 PMC5956915

[b21] Rehling P., Brandner K. & Pfanner N. Mitochondrial import and the twin-pore translocase. Nat. Rev. Mol. Cell Biol. 5, 519–30 (2004). 15232570 10.1038/nrm1426

[b22] Cieplak M., Hoang T. X. & Robbins M. O. Thermal effects in stretching of Go-like models of titin and secondary structures. Proteins: Struct. Funct. Bio. 56, 285 (2003). 10.1002/prot.20081 15211512

[b23] Tsai J., Taylor R., Chotchia C. & Gerstein M. The packing density in proteins: Standard radii and volumes. J. Mol. Biol. 290, 253 (1999). 10388571 10.1006/jmbi.1999.2829

[b24] Sułkowska J. I. & Cieplak M. Selection of optimal variants of Go-like models of proteins through studies of stretching. Biophys. J 95, 3174–3191 (2008). 18567634 10.1529/biophysj.107.127233 PMC2547460

[b25] Deutsch J. M. Biophysics software for interdisciplinary education and research. Amer. J. Phys. 82, 442–450 (2014).

[b26] Szymczak P. & Cieplak M. Stretching of proteins in a uniform flow. J. Chem. Phys. 125, 164903 (2006). 17092135 10.1063/1.2358346

[b27] Cieplak M. & Szymczak P. Protein folding in a force clamp. J. Chem. Phys. 124, 194901 (2006). 16729838 10.1063/1.2192768

[b28] Briggs M. S. & Roder H. Early hydrogen-bonding events in the folding reaction of ubiquitin. Proc. Natl. Acad. Sci. USA 89, 2017–2021 (1992). 1312711 10.1073/pnas.89.6.2017 PMC48587

[b29] Nymeyer H., Garca A. E. & Onuchic J. N. Folding funnels and frustration in off-lattice minimalist protein landscapes. Proc. Natl. Acad. Sci. USA 95, 5921–5928 (1998). 9600893 10.1073/pnas.95.11.5921 PMC34496

[b30] Sulkowska J. I. & Cieplak M. Mechanical stretching of proteins - a theoretical survey of the Protein Data Bank. J. Phys.: Condens. Matt 19, 283201 (2007).

[b31] Huang L., Kirmizialtin S. & Makarov D. Computer simulations of the translocation and unfolding of a protein pulled mechanically through a pore. J. Chem. Phys. 123, 124903 (2005). 16392523 10.1063/1.2008231

[b32] West D. K., Brockwell D. J. & Paci E. Prediction of the translocation kinetics of a protein from its mechanical properties. Biophys. J 91, L51–3 (2006). 16815903 10.1529/biophysj.106.089490 PMC1544310

[b33] Kirmizialtin S., Huang L. & Makarov D. E. Computer simulations of protein translocation. Phys. Status Solidi B 243, 2038–2047 (2006).

[b34] Huang L. & Makarov D. E. Translocation of a knotted polypeptide through a pore. J. Chem. Phys. 129, 121107 (2008). 19044999 10.1063/1.2968554

[b35] Ammenti A., Cecconi F., Marconi U. M. B. & Vulpiani A. A statistical model for translocation of structured polypeptide chains through nanopores. J. Phys. Chem. B 113, 10348–10356 (2009). 19572676 10.1021/jp900947f

[b36] Chinappi M., Cecconi F. & Casciola C. M. Computational analysis of maltose binding protein translocation. Philos. Mag. 91, 2034–2048 (2011).

[b37] Bacci M., Chinappi M., Casciola C. M. & Cecconi F. Protein translocation in narrow pores: Inferring bottlenecks from native structure topology. Phys. Rev. E 88, 022712 (2013). 10.1103/PhysRevE.88.022712 24032869

[b38] Wojciechowski M., Szymczak P., Carrión-Vázquez M. & Cieplak M. Protein unfolding by biological unfoldases: Insights from modeling. Biophys. J 107, 1661–1668 (2014). 25296319 10.1016/j.bpj.2014.07.035 PMC4190598

[b39] Makarov D. E. Computer simulations and theory of protein translocation. Acc. Chem. Res. 42, 281–289 (2008). 19072704 10.1021/ar800128x

[b40] Makarov D. E. Computational and theoretical insights into protein and peptide translocation. Protein Pept. Lett. 21, 217–226 (2014). 24370258 10.2174/09298665113209990073

[b41] Cecconi F., Bacci M. & Chinappi M. Protein transport across nanopores: A statistical mechanical perspective from coarse-grained modeling and approaches. Protein Pept Lett. 21, 227–234 (2014). 24370254 10.2174/0929866521666131227160550

[b42] Dzubiella J. Sequence-specific size, structure, and stability of tight protein knots. Biophys. J 96, 831–9 (2009). 19186124 10.1016/j.bpj.2008.10.019 PMC2716640

[b43] Maddocks J. H. & Keller J. B. Ropes in equilibrium. SIAM J. Appl. Math. 47, 1185–1200 (1987).

[b44] Bell G. I. Models for the specific adhesion of cells to cells. Science 200, 618–627 (1978). 347575 10.1126/science.347575

[b45] Hornsby C. E. & Paton R. S. It is all downhill from here. Nat. Chem. 6, 88–89 (2014). 24451579 10.1038/nchem.1852

[b46] Ess D. H. . Bifurcations on potential energy surfaces of organic reactions. Angew. Chem. Int. Ed. 47, 7592–7601 (2008). 10.1002/anie.200800918 PMC2790825 18767086

[b47] Alder N. N. & Theg S. M. Energy use by biological protein transport pathways. Trends Biochem. Sci. 28, 442–451 (2003). 12932733 10.1016/S0968-0004(03)00167-1

[b48] Rosa A., Di Ventra M. & Micheletti C. Topological jamming of spontaneously knotted polyelectrolyte chains driven through a nanopore. Phys. Rev. Lett. 109, 118301 (2012). 23005684 10.1103/PhysRevLett.109.118301

[b49] Kirmizialtin S. & Makarov D. E. Simulations of the untying of molecular friction knots between individual polymer strands. J. Chem. Phys. 128, 094901 (2008). 18331111 10.1063/1.2835605

[b50] Matthews R., Louis A. & Yeomans J. Knot-controlled ejection of a polymer from a virus capsid. Phys. Rev. Lett. 102, 088101 (2009). 19257792 10.1103/PhysRevLett.102.088101

[b51] Marenduzzo D. . DNA-DNA interactions in bacteriophage capsids are responsible for the observed DNA knotting. Proc. Natl. Acad. Sci. USA 106, 22269–22274 (2009). 20018693 10.1073/pnas.0907524106 PMC2799769

[b52] Marenduzzo D., Micheletti C., Orlandini E. & Sumners D. W. Topological friction strongly affects viral DNA ejection. Proc. Natl. Acad. Sci. USA 110, 20081–20086 (2013). 24272939 10.1073/pnas.1306601110 PMC3864349

[b53] Thomas N., Imafuku Y. & Tawada K. Molecular motors: thermodynamics and the random walk. Proc. R. Soc. Lond. Ser. B. Biol. Sci. 268, 2113–2122 (2001). 10.1098/rspb.2001.1764 PMC1088855 11600075

[b54] Wang H. & Oster G. Ratchets, power strokes, and molecular motors. Appl. Phys. A 75, 315–323 (2002).

[b55] Oster G. & Wang H. Reverse engineering a protein: the mechanochemistry of ATP synthase. Biochim. Biophys. Acta, Bioenerg 1458, 482–510 (2000). 10.1016/s0005-2728(00)00096-7 10838060

[b56] Metzler R. . Diffusion mechanisms of localised knots along a polymer. Europhys. Lett. 76, 696–702 (2006).

[b57] Tian P. & Andricioaei I. Repetitive pulling catalyzes co-translocational unfolding of barnase during import through a mitochondrial pore. J. Mol. Biol. 350, 1017–34 (2005). 15979642 10.1016/j.jmb.2005.05.035

[b58] Redner S. A guide to first-passage processes (Cambridge University Press, 2001).

[b59] Ben-Naim E., Daya Z., Vorobieff P. & Ecke R. E. Knots and random walks in vibrated granular chains. Phys. Rev. Lett. 86, 1414 (2001). 11290156 10.1103/PhysRevLett.86.1414

[b60] Luccioli S., Imparato A., Mitternacht S., Irbäck A. & Torcini A. Unfolding times for proteins in a force clamp. Phys, Rev. E 81, 010902 (2010). 10.1103/PhysRevE.81.010902 20365316

[b61] Evans E. & Ritchie K. Dynamic strength of molecular adhesion bonds. Biophys. J. 72, 1541–1555 (1997). 9083660 10.1016/S0006-3495(97)78802-7 PMC1184350

[b62] Szymczak P. & Cieplak M. Stretching of proteins in a force-clamp. J. Phys. Condens. Matt. 18, L21 (2006).

[b63] West D. K., Brockwell D. J., Olmsted P. D., Radford S. E. & Paci E. Mechanical resistance of proteins explained using simple molecular models. Biophys. J 90, 287–297 (2006). 16214858 10.1529/biophysj.105.071035 PMC1367027

[b64] Hurt E. C. & van Loon A. P. How proteins find mitochondria and intramitochondrial compartments. Trends Biochem. Sci. 11, 204–207 (1986).

[b65] Wilcox A. J., Choy J., Bustamante C. & Matouschek A. Effect of protein structure on mitochondrial import. Proc. Natl. Acad. Sci. USA 102, 15435–15440 (2005). 16230614 10.1073/pnas.0507324102 PMC1266127

[b66] Sato T., Esaki M., Fernandez J. M. & Endo T. Comparison of the protein-unfolding pathways between mitochondrial protein import and atomic-force microscopy measurements. Proc. Natl. Acad. Sci. USA 102, 17999–8004 (2005). 16326810 10.1073/pnas.0504495102 PMC1312372

[b67] Schneider G. F. & Dekker C. DNA sequencing with nanopores. Nat. Biotechnol. 30, 326–328 (2012). 22491281 10.1038/nbt.2181

[b68] Bayley H. Nanopore sequencing: From imagination to reality. Clin. Chem. 61, 25–31 (2015). 25477535 10.1373/clinchem.2014.223016 PMC4404466

[b69] Plesa C. . Observation of DNA knots using solid-state nanopores. Biophys. J 108, 166a (2015).

[b70] Li W. . Single protein molecule detection by glass nanopores. ACS nano 7, 4129–4134 (2013). 23607870 10.1021/nn4004567

[b71] Plesa C. . Fast translocation of proteins through solid state nanopores. Nano Lett. 13, 658–663 (2013). 23343345 10.1021/nl3042678 PMC4151282

[b72] Rosen C. B., Rodriguez-Larrea D. & Bayley H. Single-molecule site-specific detection of protein phosphorylation with a nanopore. Nat. Biotechnol. 32, 179–181 (2014). 24441471 10.1038/nbt.2799 PMC4391620

[b73] Millar R. B. Maximum likelihood estimation and inference: with examples in R, SAS and ADMB (John Wiley & Sons, 2011).

