# Supplementary material for: Periodic forces trigger knot untying during translocation of knotted proteins
Source: Sci Rep. 2016 Mar 21;6:21702. doi: 10.1038/srep21702 (PMC4800218; doi:10.1038/srep21702)
Supplement: Supplementary Information [file srep21702-s3.docx]

The supplementary material contains two videos:

- movieS1.avi - presenting the results of computer simulations of protein translocation through the pore under the action of a constant force. The particular protein modeled here is E. coli YbeA methyltransferaze (pdb code: 1ns5) and the trajectory corresponds to that shown in Fig,. 2a in the main text.

- movieS2.avi - same as in movie1, but for repetitive force (corresponding to Fig. 5 in the main text)
